# Seepage evolution characteristics and water inrush mechanism in collapse column under mining influence

**DOI:** 10.1038/s41598-024-54180-z

**Published:** 2024-03-11

**Authors:** Wu Yongjiang, Cao Zhengzheng, Li Zhenhua, Du Feng, Wang Wenqiang, Zhai Minglei, Hong Zijie, Xue Yi

**Affiliations:** 1 International Joint Research Laboratory of Henan Province for Underground Space Development and Disaster Prevention, School of Civil Engineering, Henan Polytechnic University, Jiaozuo, 454000 Henan China; 2 Henan Mine Water Disaster Prevention and Control and Water Resources Utilization Engineering Technology Research Center, Henan Polytechnic University, Jiaozuo, 454000 Henan China; 3 Collaborative Innovation Center of Coal Work Safety and Clean High Efficiency Utilization, Jiaozuo, 454000 Henan China; 4 School of Civil Engineering and Architecture, Xi’an University of Technology, Xi’an, 710048 China

**Keywords:** Collapse column, Seepage evolution, Water inrush disaster, Coupled mechanical modeling, Qianjin coal mine, Coal, Civil engineering

## Abstract

To obtain the seepage evolution rule and water inrush mechanism of the collapse column, a multi-field coupled mechanical model for water inrush disasters caused by the collapse column is established in this paper, on the basis of the specific engineering conditions of the 1908 working face in the Qianjin coal mine. The mechanical model is composed of internal column elements within the collapse column and surrounding rock masses. The research focuses on the seepage evolution rule in the roof collapse column under different mining conditions and investigates the permeation instability mechanism of collapse column based on the transition of flow state. The research results indicate that the seepage pathway evolves continuously, ultimately forming a channel for water inrush, as the working face advances towards the collapse column. Besides, the water inflow increases rapidly when the working face advances 100 m, then gradually stabilizes, indicating that the seepage channel entry of the collapse column is in a stable stage. Meanwhile, mass loss in the collapse column gradually moves upward. the collapse column remains stable as a whole in the initial stage of water flow, with a small permeability, exhibiting linear flow. As time steps increases, particle loss in collapse column gradually extends to the upper part, forming a stable seepage channel. The flow velocity shows fluctuations with a slow declining trend over time.

## Introduction

In the karst mining areas of Southwest China, extensive exposure of bedrock is prevalent, driven by the extraction of coal resources. The resultant decline in ecological water levels has triggered the development of rocky desertification in the region. Southern coalfields in China, characterized by the abundant presence of roof collapse column due to geological strata conditions, exhibit dynamic water conduction within these columns during coal mining operations. The interaction between working face excavation and overlying aquifers activates water conduits in the roof collapse column. Particulate matter within the collapse column migrates and is lost with the flowing water, leading to permeation instability and the initiation of water inrush disasters. These phenomena significantly influence the safety and efficiency of mine operations, and lead to major safety incidents such as flooding due to water inrush.

Scholars both domestically and internationally have conducted a series of theoretical studies on water inrush from roof collapse column, achieving a significant progress. Regarding structural failure, based on Griffith's theory, Coulomb's criterion, and the limit equilibrium theory, Xu et al.^1^ derived the mechanism and criteria for the activation of water conduits in collapse column. By employing damage mechanics and fluid mechanics theories, Wang et al.^2^ investigated the coupled mechanisms of seepage-fracture-damage in mine water inrush, revealing the evolving patterns of surrounding rock seepage fields and column fracture damage during excavation. Wei et al.^3^ proposed a mechanical model for collapse column under negative and positive pressures, and established a predictive system for collapse column instability to guide on-site practices. Song et al.^4^ found that the stress in a thick-walled elliptical cylinder reaches its maximum value at the endpoint of the inner wall's major axis and its minimum value at the minor axis. In the field of fluid–solid coupling research, Terzaghi^5^ was among the first scholars to investigate fluid–solid coupling, introducing the concept of effective stress and establishing a one-dimensional consolidation model. By building upon Terzaghi's work, Biot^6,7^ further explored the relationship between pore pressure and porous media. Based on the principle of effective stress in porous media, Li et al.^8^ conducted a detailed analysis of the physical properties of fluid–solid coupling seepage, and established a comprehensive saturated porous media fluid–solid coupling model. By considering the heterogeneity of fractured rock in collapse column, Yao et al.^9^ studied the water inrush process of collapse column under the loss of fill particles, revealing the mechanism of water inrush from collapse column under stress-seepage coupling. Wang et al.^10^ established a physical model of upper and lower mining in a test mine, and analyzed the dynamic evolution and mechanism of karst roof water inrush under different mining sequences. By studying the influence of liquid nitrogen fracturing on granite, Wang et al.^11,12^ showed that the cyclic application of liquid nitrogen cooling promoted the formation of internal fractures in granite and its impact on the weakening characteristics of the mechanical properties of granite. Weile et al.^13^ studied the micropore characteristics of remolded loess and undisturbed loess at different resting periods, indicating that the prepared loess samples have obvious thixotropy, which has certain guiding significance for improving the geotechnical properties. Xue et al.^14^ studied the fracturing effect of water-cooled impact on high-temperature rocks, indicating that water-cooled impact can significantly reduce fracture initiation pressure and induce more secondary fractures.

In the realm of numerical simulations for water inrush from roof collapse column, previous researchers have employed various numerical computation software to conduct simulations and simulations under different mining conditions. This has resulted in numerous research achievements regarding the evolution characteristics of seepage fields in collapse column and the entire process of water inrush. Liu et al.^15^ utilized the finite element analysis software ANSYS, and investigated the distribution patterns of coal seam floor deformation, water pressure, and stress when the working face approached or moved away from the collapse column. Subsequently, they delved into the water inrush mechanism of collapse column. By employing FLAC3D software, Yin et al.^16,17^ simulated and analyzed the water inrush mechanism of the coal seam floor collapse column as the mining face advanced. Li et al.^18^ used the RFPA2D numerical simulation software, and obtained the influence of confined water pressure and the development height of collapse column on the delay time of water inrush. By employed FLAC3D numerical software, Li et al.^19^ studied the risk of water inrush from collapse column under mining influence, indicating a significant water inrush risk when the working face advanced to a distance of 10 m from the collapse column. Yao et al.^20^ utilized COMSOL Multiphysics software to solve a coupled model for deformation, seepage, and erosion in the water inrush process from collapse column on the coal seam floor. Zhengzheng et al.^21,22^ established a numerical model of slurry diffusion in veneer cracks, and further studied the diffusion law of grouting slurry in cracks under different rheological indexes and different consistency indexes. Liu et al.^23^ conducted a numerical study on the multi-physical field coupling mechanism in the process of microwave thermal recovery of shale gas, which provided a necessary theoretical guidance for the field application of microwave thermal recovery of shale gas.

Presently, research work on the water inrush disaster mechanisms of collapse column predominantly focuses on the internal column seepage evolution within the fractured rock mass^24^. However, a more in-depth exploration of the research on collapse column bodies considering the influence of external surrounding rock has not been carried out. Taking the 1908 working face in the Qianjin coal mine as a case study, this paper establishes a multi-field coupled mechanical model for water inrush from collapse column, consisting of both the internal column elements and external surrounding rock. By investigating the evolution patterns of seepage in the roof collapse column under different mining conditions and the mechanism of permeation instability leading to disasters based on flow state transition, the mechanisms behind water inrush from collapse column in the southwestern mining regions is revealed.

## Stress-damage-seepage coupled model of water inrush in collapse column

### Mechanical model of variable-mass seepage inside collapse column

(1) Basic assumptions

In order to establish a mathematical model for water inrush from collapse column considering fluid seepage, particle migration, and pore evolution, the following assumptions are made: (1) The solid skeleton of the collapse column can be approximated as a dual medium consisting of pores and fractures; (2) Fluid movement within the collapse column adheres to Darcy's law of permeability; (3) The velocity of suspended particles is approximately equal to the fluid velocity; (4) The rock mass within the collapse column is loosely fragmented, and the influence of stress on the permeability of the collapse column is neglected; (5) The volume concentration of particles in matrix pores and fractures is approximately equal; (6) The impact of particles in the fluid on the permeability characteristics of the fluid is neglected.

(2) Basic definition

The collapse column characteristic unit is composed of three parts, containing the solid skeleton, fluid, and suspended particles in the fluid. Among these, the volume concentration of suspended particles is denoted as *C*, and the density of particles is represented by $\rho_{s}$. Assuming the side length of the matrix block is *a*, the fracture width is *b*, and $b \ll a$, the volume of the unit element is given by

1 $$V = (a + b)^{3} \cong a^{3}$$

Assuming the porosity of the matrix block is denoted as $\phi$, the void ratio of the unit element is given by

2 $$\varphi = \frac{{(a + b)^{3} - a^{3} }}{{(a + b)^{3} }} + \phi \cong \frac{3b}{a} + \phi$$

Assuming the Darcy seepage velocity is represented by ${q}_{i}$, the true velocity of the fluid is given by

3 $$v_{i} = \frac{{q_{i} }}{\varphi }\quad (i = 1,2,3)$$

(3) Particle mass conservation equation

**Figure 1:**
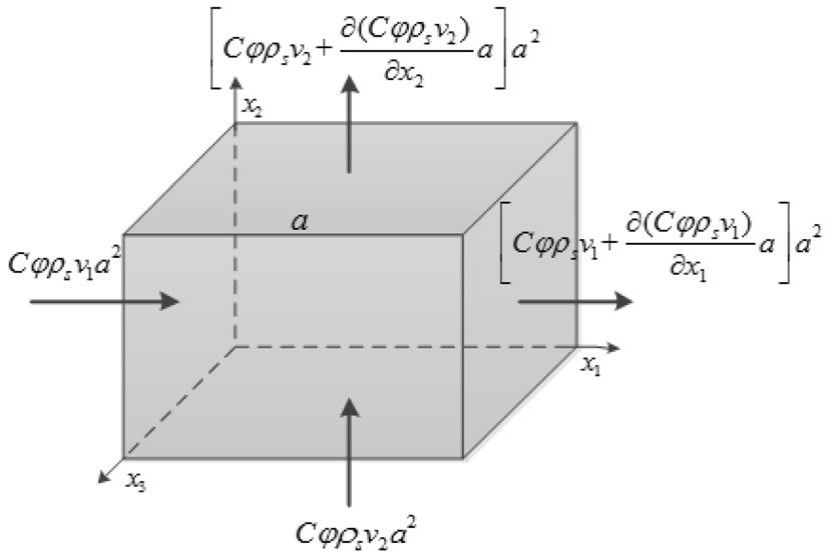
Characteristic micro-element of a collapse column.

The migration of particles within the fill material of collapse column under erosion can be considered as the combined effect of convection and diffusion, with a specific focus on convection. As illustrated in Fig. 1, representing a characteristic micro-element of the collapse column, the mass of particles entering the unit element per unit time due to the effects of convection and diffusion in the $x_{i}$ $(i = 1,2,3)$ direction can be expressed as follows

4 $$- \frac{{\partial (C\varphi \rho_{s} v_{i} )}}{{\partial x_{i} }}a^{3}$$

5 $$\frac{\partial }{{\partial x_{i} }}\left( {D\frac{{\partial (C\varphi \rho_{s} )}}{{\partial x_{i} }}} \right)a^{3}$$

In the *x*-direction, the mass of particles entering the unit element per unit time under the combined influence of convection and diffusion is given by

6 $$- \frac{{\partial (C\varphi \rho_{s} v_{i} )}}{{\partial x_{i} }}a^{3} + \frac{\partial }{{\partial x_{i} }}\left( {D\frac{{\partial (C\varphi \rho_{s} )}}{{\partial x_{i} }}} \right)a^{3}$$

According to the principle of mass conservation, the inflow mass accounts for the increase in mass within the unit element volume per unit time.

7 $$\frac{\partial }{\partial t}(C\varphi \rho_{s} )a^{3} - \dot{m}a^{3}$$

where $\dot{m}$ represents the mass of particles eroded from the unit element into the fluid per unit time and unit volume. The relationship between $\dot{m}$, *a* and *b* satisfy the following equation

8 $$\dot{m} = \frac{\partial \varphi }{{\partial t}}\rho_{s}$$

If the diffusion effect of particles is neglected, the governing equation for particle motion is

9 $$\left( {\frac{3}{a}\frac{\partial (bC)}{{\partial t}} + \frac{\partial (C\phi )}{{\partial t}}} \right) + \nabla \cdot (C\vec{q}) = \left( {\frac{3}{a}\frac{\partial b}{{\partial t}} + \frac{\partial \phi }{{\partial t}}} \right)$$

(4) Conservation equation of fluid mass

10 $$\left( {\frac{3}{a}\frac{{\partial \left( {b(1 - C)} \right)}}{\partial t} + \frac{{\partial \left( {(1 - C)\phi } \right)}}{\partial t}} \right) + \nabla \cdot \left( {(1 - C)\vec{q}} \right) = 0$$

(5) Pore evolution governing equation

Sakthivadivel and Irmay, as well as Sakthivadivel, conducted research on the erosion of porous media by using theoretical analysis and experimental methods. I. Vardoulakis et al. summarized previous research findings on the erosion of porous media, providing the governing equation for pore evolution under particle migration in the context of porous media erosion.

11 $$\frac{\partial \phi }{{\partial t}} = \lambda_{1} \rho_{s} (\phi_{\max } - \phi )c\parallel q\parallel$$

Similarly, the evolution equation for fractures in dual-porosity media under erosion can be expressed as

12 $$\frac{\partial b}{{\partial t}} = \lambda_{2} \rho_{s} (b_{\max } - b)c\parallel q\parallel$$

The above equation indicates that the change in porosity caused by erosion is directly proportional to the particle concentration and the seepage velocity, where $\lambda_{1}$ and $\lambda_{2}$ are constants, and $\parallel q\parallel { = }\sqrt {q_{1}^{2} + q_{2}^{2} + q_{3}^{2} }$ is the absolute value of the fluid seepage velocity.

(6) Conservation equation of fluid mass

The motion equation of the fluid can be expressed by Darcy's law as follows

13 $$\vec{q} = - \frac{k}{\eta }(\nabla p + \rho_{f} g\nabla z)$$

where $\vec{q}$ represents the Darcy velocity of the fluid (m/s); $k$ is the permeability of the unit element (m2); $\mu$ is the dynamic viscosity of the fluid (${\text{Pa}} \cdot {\text{s}}$); $p$ is the pore pressure (Pa); $\rho$ is the density of the fluid (${\text{kg/m}}^{{3}}$); and $\nabla z$ is the unit vector in the direction of gravitational force.

(7) Porosity–permeability relationship equation

14 $$k_{e} = k_{m} + k_{{_{f} }} = k_{m0} \left( {\frac{{\phi_{m} }}{{\phi_{m0} }}} \right)^{3} \left( {\frac{{1 - \phi_{m0} }}{{1 - \phi_{m} }}} \right)^{2} + \frac{{b^{3} }}{12a}$$

**Figure 2:**
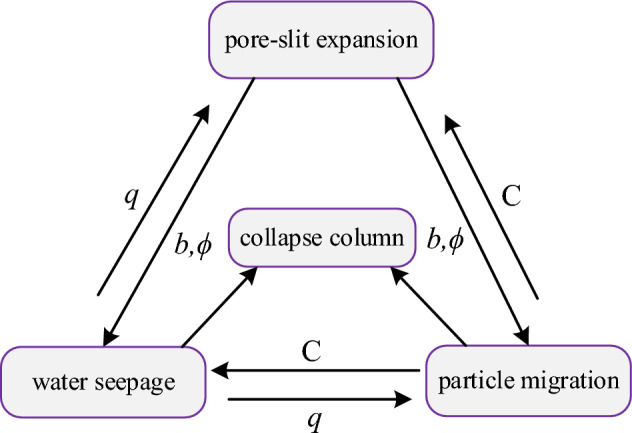
Coupling relationships of the model equations.

Taking into account the six Eqs. (9) to (14) and the six unknowns $\phi$, $b$, $k_{e}$, $C$, $p$, and $\vec{q}$, the system of equations are in closed state, forming a comprehensive mechanical model for water inrush from collapse column under erosion. The coupling relationships among the model equations are illustrated in Fig. 2.

### Damage constitutive mechanical model of external rock mass in collapse column

#### Rock mechanical model based on micro-unit damage evolution

(1) Ontological model

Rock is a complex material containing joint fissures, and under external forces, the stress–strain relationship exhibits non-linearity. Therefore, it is necessary to establish a rock damage constitutive model to analyze the mechanical properties of rock during the damage process. According to the Lemaitre equivalent strain hypothesis, the fundamental relationship of the rock damage constitutive model is given by

15 $$\sigma^{*} = \frac{\sigma }{1 - D}$$

In the equation, $\sigma^{*}$ represents the effective stress of the undamaged part of the rock, $\sigma$ is the nominal stress, and *D* is the damage variable.

To describe the heterogeneity of rock material parameters such as elastic modulus, strength, permeability, etc., it is necessary to assign corresponding mechanical parameters to micro-elements. Assuming that they follow a Weibull distribution, the probability density function is given by

16 $$f(x) = \frac{m}{n}\left( \frac{x}{n} \right)^{m - 1} \exp \left[ { - \left( \frac{x}{n} \right)^{m} } \right]$$

where *m* represents the degree of rock in-homogeneity, *x* is the random distribution variable of rock micro-mechanical parameters, *n* is the average value of rock micro-mechanical parameters.

Assuming that the rock micro-element damage probability obeys the probability density function *f*(*x*), the damage variable is

17 $$D = 1 - \exp \left[ { - \left( \frac{x}{n} \right)^{m} } \right]$$

The stress–strain relationship of the undamaged micro-elements of the rock satisfies Hooke's law in the principal stress state.

18 $$\varepsilon_{i} = \frac{1}{E}\left[ {\sigma_{i}^{*} - v\left( {\sigma_{j}^{*} + \sigma_{k}^{*} } \right)} \right]$$

where $v$ is Poisson's ratio, $\varepsilon$ is strain, *E* is elastic modulus, and *i*, *j*, *k* is 1, 2, 3 and unequal.

According to Eqs. (14) and (15), the micro-elementary damage ontological model of rock based on Weibull distribution is obtained, and the second principal stress is equal to the third principal stress in conventional triaxial compression test of rock, i.e., the final equation is transformed into

19 $$\sigma_{1} = E\varepsilon_{1} \exp \left[ { - \left( \frac{x}{n} \right)^{m} } \right] + 2v\sigma_{3}$$

According to the effective stress principle

20 $$\sigma_{ij} = \sigma^{\prime}_{ij} + p_{w} \delta_{ij}$$

The intrinsic model of rock seepage damage based on Weibull distribution is obtained,

21 $$\sigma_{1} = E\varepsilon_{1} \exp \left[ { - \left( \frac{x}{n} \right)^{m} } \right] + 2v\sigma_{3} + (1 - 2v)p_{w}$$

#### Multi-coupled control equations

(1) Deformation equation

Assuming that each stress component on the rock micro-element satisfies the hydro-static equilibrium condition

22 $$\sigma_{ij,j} + f_{i} = 0$$

where $\sigma_{ij.j}$ is the component of the unit stress tensor and *f*_*i*_ is the component of the body force.

Rock micro-elements are elastically deformed by external forces, and the strains and displacements satisfy the geometric equations

23 $$\varepsilon_{ij} = \frac{1}{2}(u_{i,j} + u_{j,i} )$$

where $\varepsilon_{ij}$ is the strain tensor *u*_*i*_ and *u*_*j*_ are the components of the displacement in the *i* and *j* directions, respectively.

(2) Flow equation

Seepage field control equation is

24 $$S\frac{\partial p}{{\partial t}} + \nabla \cdot \left( { - \frac{k}{\mu }\nabla p} \right) = Q_{m}$$

Assuming that the rock skeleton produces elastic deformation, and considering the effect of pore water pressure on the volumetric deformation of the skeleton, a porosity evolution equation is established to describe the change of the porosity of the rock under constant temperature conditions.

25 $$\phi_{s} = \alpha - (\alpha - \phi_{s0} )\exp \left( { - \varepsilon_{v} - \frac{\nabla p}{{K_{s} }}} \right)$$

where $\alpha$ is the Biot coefficient, $\phi_{s0}$ is the initial porosity of the rock, and *K*_*s*_ is the bulk modulus of the rock particles.

When considering the effect of damage on rock permeability, the following relationship is satisfied

26 $$k = k_{0} \left( {\frac{{\phi_{s} }}{{\phi_{s0} }}} \right)^{3} \exp \left( {\alpha_{k} D} \right)$$

where $\alpha_{k}$ is the coefficient of influence of damage on permeability, taken as 5.0.

Bringing Eq. (25) into the cubic law (26) leads to a model for the evolution of the permeability coefficient.

27 $$k = k_{0} \left[ {\frac{{\alpha (1 + \varepsilon_{v} ) - (\alpha - \phi_{s0} )\left( {1 - \frac{\nabla p}{{K_{s} }}} \right)}}{{\phi_{s} (1 + \varepsilon_{v} )}}} \right]^{3}$$

**Figure 3:**
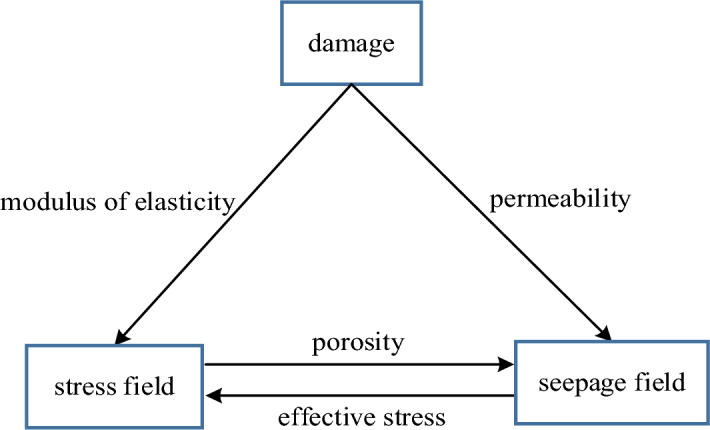
Multi-field coupling relationship of rock damage ontological model.

The rock damage evolution ontological model of stress-seepage coupling can be constructed by the above theoretical formulation, and the relationship between the stress field, seepage field, and damage is shown in Fig. 3.

## Multi-field coupled numerical model of water inrush disaster in collapse column

The collapse column is situated beneath the aquifer, typically residing in deeper geological strata. The aquifer, serving as the overlying layer, provides moisture that contributes to the formation of the collapse column through dissolution processes. The development of the collapse column influence the structure and functionality of the overlying aquifer. The cause of water inrush in collapse column is attributed to the disturbance of the equilibrium between surrounding rock and water during coal mining. Under the influence of disturbances caused by mining, numerous random fractures gradually develop within the collapse column. As a result of water seepage, the fill particles within these fractures are continuously transported, leading to the further expansion of the fractures. The liquid carrying fine particles exerts erosive and abrasive effects on the rock mass, triggering the continued expansion and connection of fractures. Ultimately, a stable water passage is formed.

### Numerical simulation model

**Figure 4:**
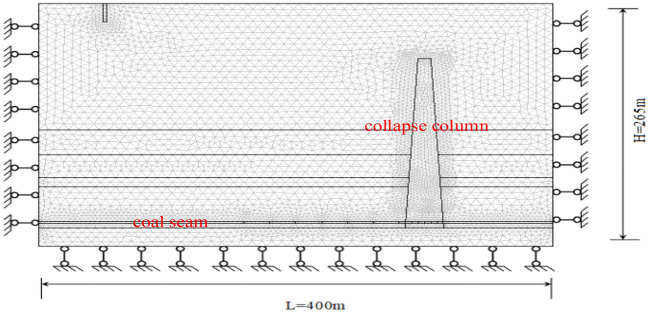
Numerical calculation model.

Based on the geological conditions detected in the 1908 working face of the Qianjin coal mine, the geometric dimensions of the calculation model for the coal seam working face containing a collapse column are set at 400 × 200 × 265 m. The cross-sectional view of the model is shown in the Fig. 4, with the 2 m coal seam thickness.

The working face advances from the left side of the model to the right in a one-cut full height manner. The collapse column is located in the middle of the model, with a height of 185 m, a bottom diameter of 30 m, and a top diameter of 10 m. The model bottom is constrained in the vertical direction, and the left and right sides are constrained in the horizontal direction, while the upper part of the model is an open boundary. For the permeability of the mining area, the concave depression in the upper left part of the model serves as a water pressure source. After the advancement of the working face, the water pressure in the goaf becomes 0, and the remaining boundaries are impermeable. The initial moment is set at the time of coal excavation, and the Mohr–Coulomb failure criterion is employed.

### Numerical simulation results

(1) Evolutionary cloud maps and seepage vectors of the water inrush channel

**Figure 5:**
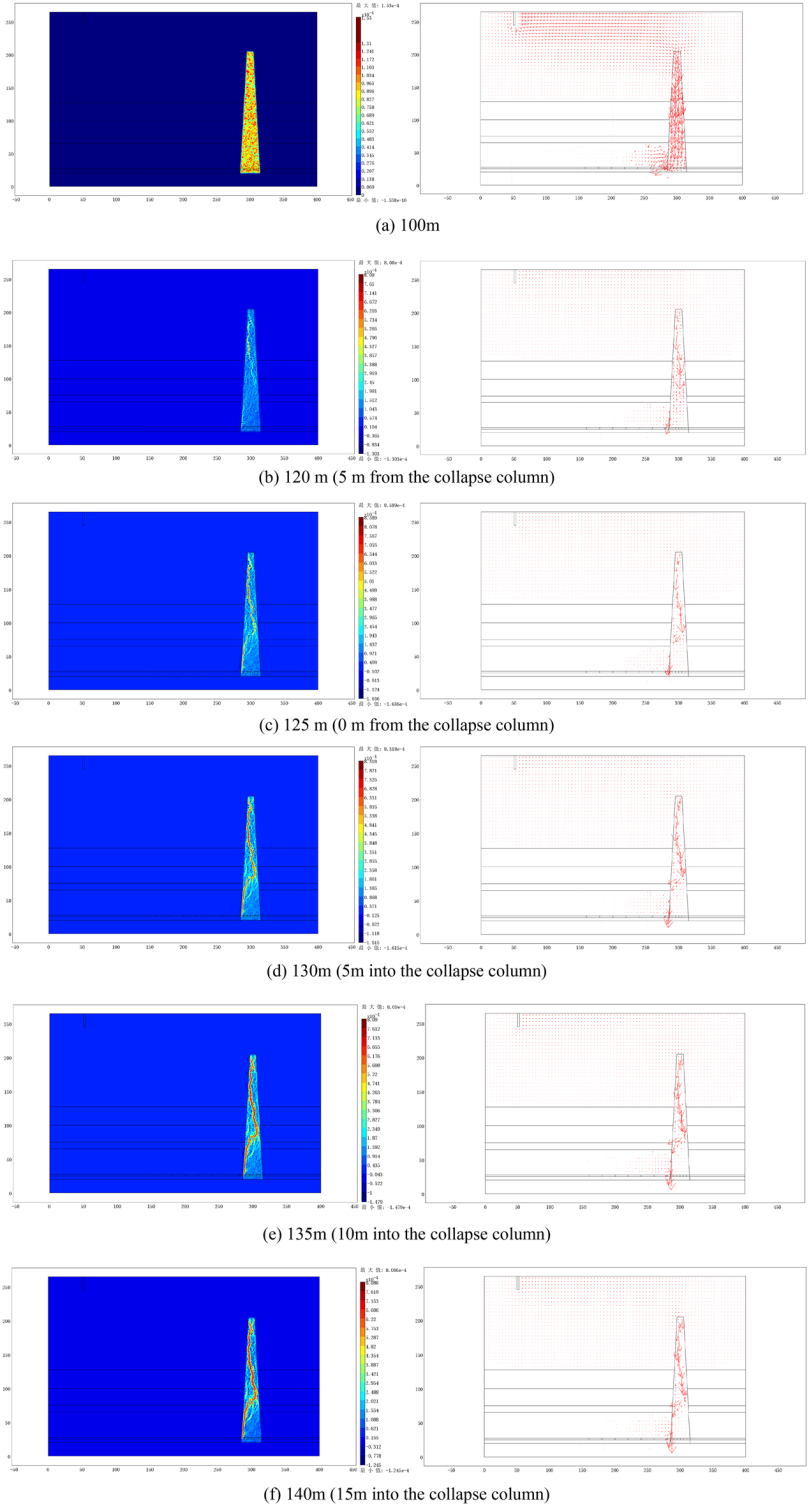
Evolution of water inrush channels and seepage vectors.

From Fig. 5, it can be observed that as the working face continuously advances toward the collapse column, the seepage channels evolve continuously, ultimately forming a water inrush channel. Before the working face is 25 m away from the collapse column, there is no significant change in the fractures inside the collapse column. When the working face advances to within 5 m of the collapse column, the seepage channel inside the collapse column begins to form. By the time the working face advances to 15 m inside the collapse column, the seepage channel inside the collapse column has fully formed. Through the analysis of seepage vectors, the formation path of the water inrush channel can be observed. It initiates from the concave depression in the upper left part of the model, diagonally moves to the lower right part, enters through the top of the collapse column, undergoes some curvature within the collapse column, and finally enters the working face from the left side at the bottom of the collapse column.

**Figure 6:**
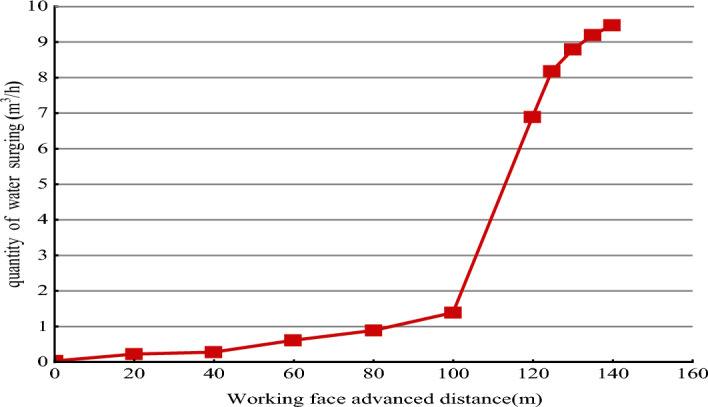
Change curve of water influx with advancement of working face.

From Fig. 6, it can be observed that with the increase in the advancement distance of the working face, before reaching 100 m, the water inflow from the collapse column is relatively small, approximately 0 to 1 m^3^/h. However, after advancing to 100 m, due to the influence of mining activities, the internal fractured rock mass within the collapse column experiences a seepage-erosion gushing water effect, leading to a rapid increase in the water inflow to 9.8 m^3^/h. As the working face continues to advance, the water inflow gradually stabilizes, indicating that the seepage channels within the collapse column enter a stable stage. This process of water inflow variation is consistent with on-site monitoring, further confirming the rationality and scientific validity of the collapse column mass change gushing water model.

## Water inrush mechanism induced by permeability destabilization of collapse column based on the flow state transition

From the microscopic point of view, the variable mass seepage process in collapse column is the erosion, dissolution, abrasion and other interactions among broken rock, fluid medium, fine particles, leading to the change in porosity and permeability, and this process is inextricably linked with the time. When the pressure gradient is larger, the completion of the flow transition time is shorter. When the pressure gradient is smaller, the process appears slow, but ultimately still shows the change in the flow transition.

In order to analyze the flow state of the collapse column with time from non-Darcy high-velocity flow into Navier–Stokes turbulent flow during the mass change process in detail, the collapse column is divided into six steps from the bottom to the top, namely, 30 m, 60 m, 90 m, 120 m, 150 m, and 180 m, which corresponds to the state of the collapse column at six consecutive time intervals, and the collapse column region in the upper part of the interface of each step satisfies the porous in each step, the upper collapse column region of the interface satisfies the porous Brinkman equation with Forchheimer's modification, and the lower collapse column region of the interface satisfies the Navier–Stokes turbulence equation.

**Figure 7:**
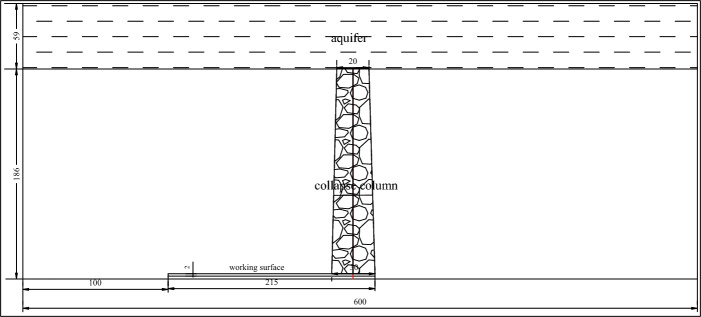
Numerical simulation of different time steps of water inrush in collapse column.

The numerical calculation model is shown in Fig. 7. The aquifer permeability is 2.1 × 10^−12^, the porosity is 0.14, the permeability of collapse column is 2.1 × 10^−10^, the porosity is 0.348. The model aquifer boundary is a fixed pressure boundary, the boundary pressure is 4.1 MPa, and the outlet is one atmosphere, and the free triangle mesh is used to divide into 3098 cells.

**Figure 8:**
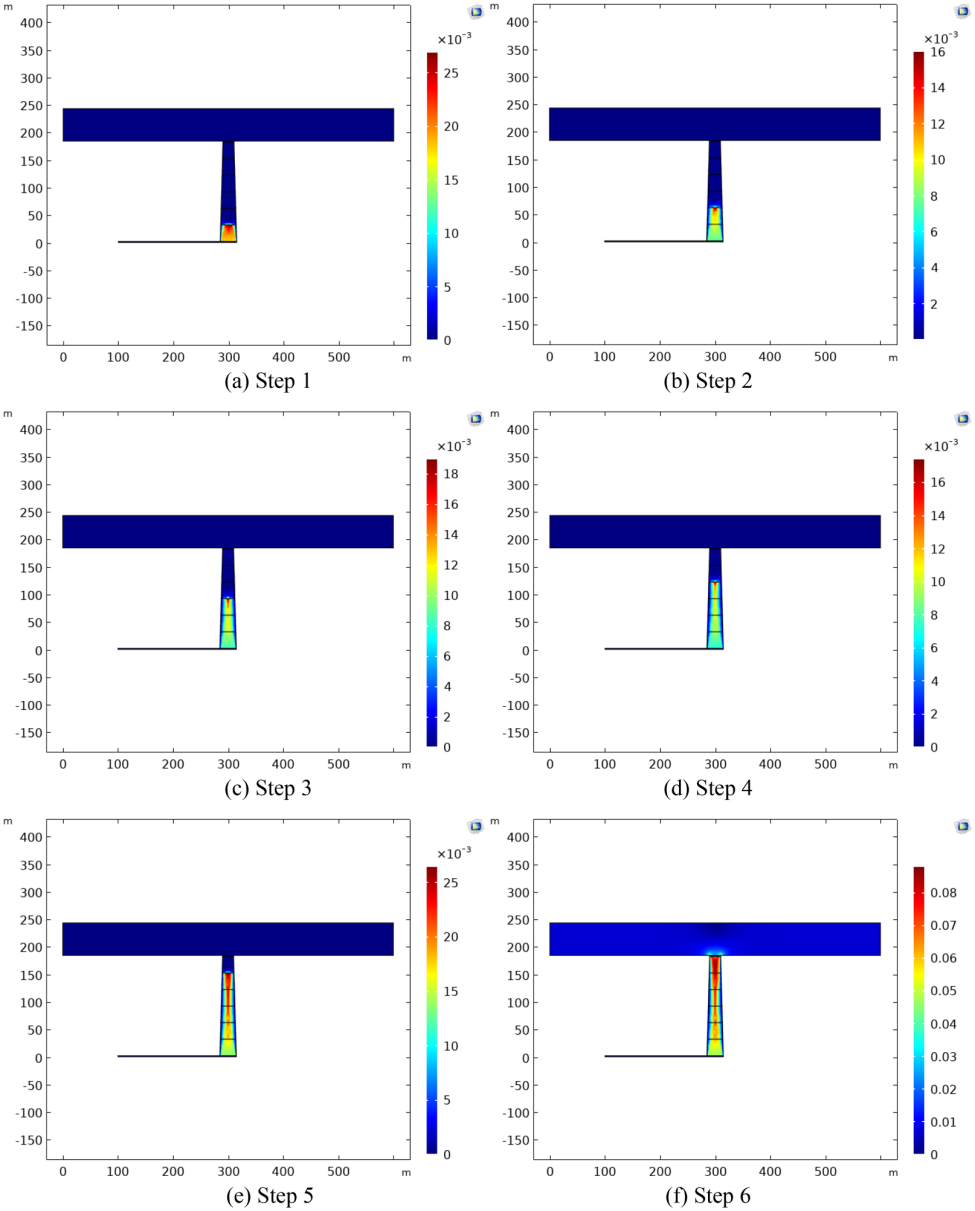
Flow velocity distribution at different time steps.

By observing the velocity distributions corresponding to the six time steps, shown in Fig. 8, it is evident that the velocity of the collapse column can be divided into two parts. Through analysis of the equations corresponding to each time step and the velocity distributions, the region satisfying the Brinkman equation corrected by Forchheimer for porous areas exhibits significantly faster velocities than the region satisfying the Navier–Stokes turbulent flow equation. With the progression of time steps, the process of mass transition intensifies, and the interface gradually moves upward. In this process, as the mass transition proceeds gradually, the region satisfying the N-S turbulent flow equation gradually increases, while the porous medium region gradually decreases. The velocities increase steadily. When a sufficient amount of time has elapsed, the entire collapse column region satisfies the Navier–Stokes turbulent flow equation, leading to a pronounced transition in flow state, and the velocity and risk of water influx further escalate.

**Figure 9:**
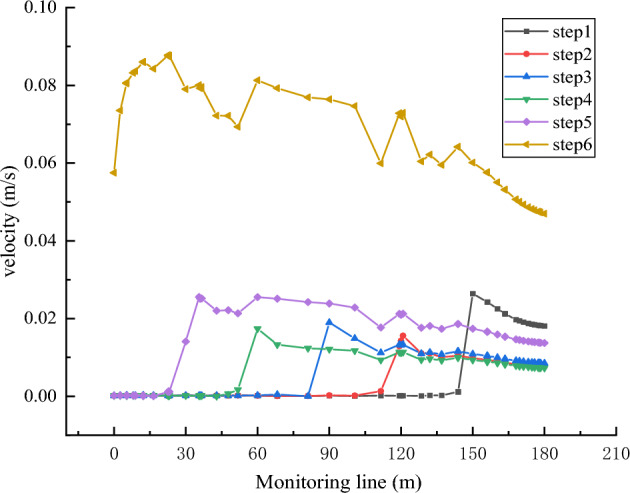
Velocity profile at different time steps.

By observing the velocity curve in Fig. 9, it is obvious that the velocity remains constant in the first five steps until it reaches the interface between the two equations along monitoring line C-D, where the velocity undergoes a sudden change. It rapidly increases, gradually declines to a constant value, and in the sixth step, it rises from 0.0575 m/s to 0.08607 m/s. Subsequently, it exhibits fluctuations and an overall decreasing trend, indicating continuous mass loss in the collapse column, destabilizing progressively from the bottom to the top. The water influx velocity in the first step is significantly higher than in steps 2, 3, 4, and 5, indicating that the initial water influx velocity is notably larger, and the initial phase is relatively vulnerable. Therefore, the key to preventing disasters such as water influx in the collapse column lies in sharp early monitoring and timely management. When the collapse column approaches complete penetration, the water influx speed is generally fast, far exceeding the first five steps. Therefore, immediate measures should be taken once water influx occurs to prevent excessive mass loss in the collapse column, avoiding complete penetration with the aquifer, which can lead to rapid water influx.

**Figure 10:**
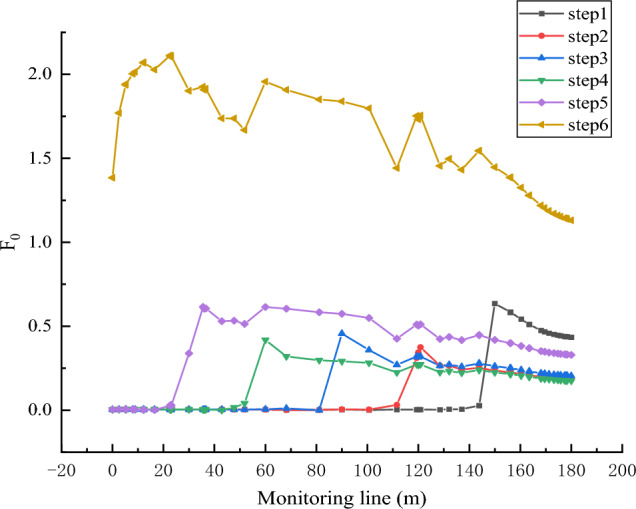
Plot of Forchheimer coefficients for different time steps.

**Figure 11:**
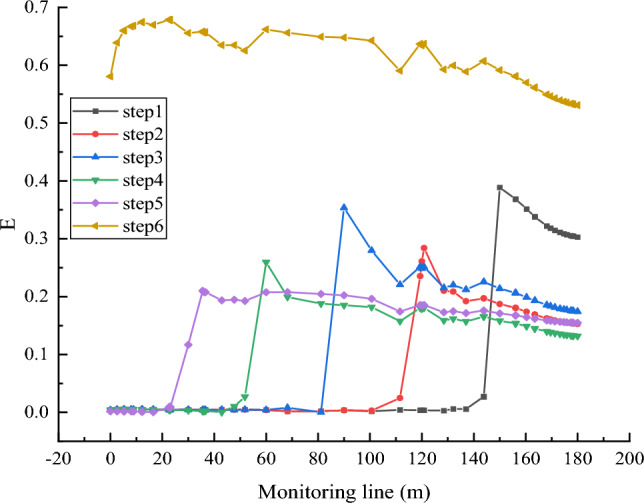
Non-Darcy effect at different time steps *E.*

Through the observation of Figs. 10 and 11, when the collapse column has not reached the instability interface, the Forchheimer number (*F*_0_) and the non-Darcy effect (*E*) are minimal, approaching zero. This indicates that the fluid flow inside the collapse column exhibits relatively weak nonlinear characteristics at this stage, leaning towards linear flow. However, once the collapse column reaches the instability interface, *F*_0_ and non-Darcy effect *E* rapidly increase. This suggests that in the event of water influx, the velocity is many times that of the flow velocity within the collapse column. At the interface and its vicinity, *F*_0_ significantly increases but remains below 1 in the first five steps, indicating that under local mass loss conditions, the fluid entering the collapse column transitions gradually from linear flow to nonlinear flow. However, the degree of non-linearity is low and does not reach the level of turbulence. This implies that mass loss creates local seepage channels, and the velocity is not a constant but fluctuates, encountering hindrance during flow and exhibiting a slow declining trend. When the collapse column almost entirely satisfies the Navier–Stokes equations over time, both the Forchheimer number and the non-Darcy effect significantly increase. This signifies a sharp rise in seepage velocity, substantial nonlinear flow, and a high likelihood of severe water influx.

## Conclusions

In response to water inrush disaster in the collapse column induced by mining activities, this paper proposes a multi-field coupled mechanical model for water inrush disaster in the collapse column, consisting of the internal columnar structure of the collapse column and the surrounding rock. Numerical simulations are conducted to investigate the evolution of seepage in the roof collapse column under different mining conditions, and the water inrush mechanism induced by the permeability instability of the collapse column based on flow transition. The study systematically elucidates the evolution of seepage in the collapse column and the mechanism of water inrush disaster under mining influence. The following conclusions are obtained.

1. Continuous medium mechanical methods are employed to investigate the loss of fillings and the evolution of permeability in the process of fractured rock mass seepage. A dynamic model for the coupled flow and mass transfer in fractured rock mass seepage is established. Based on the principles of elasticity, damage theory, and seepage theory, a damage constitutive model for rock micro-element is constructed. By linking the stress field, seepage field, and damage field, the dynamic model reveals the seepage mutation mechanism in fractured rock mass from the perspectives of mass transfer and fluid–solid coupling.
2. Through numerical simulation, the evolution process of the seepage channel in the collapse column under mining-induced stress and the variation in water inflow are reproduced. As the advancing distance of the working face increases, the internal fractures of the collapse column gradually enlarges, forming a seepage channel. At the same time, the water inflow exhibits a slow-rapid-stable three-stage pattern. This variation process closely aligns with on-site monitoring, further confirming the rationality and scientific validity of the model for mass transfer water inrush in fractured rock masses within collapse column.
3. Through the observation of velocity cloud maps, the mass loss in the roof collapse column gradually moves upward. In the initial stage of water flow, the collapse column remains stable as a whole, with a small permeability and linear flow. As the time steps increases, the particle loss gradually expands to the upper part, forming a stable seepage channel, and the flow velocity exhibits fluctuations with a slow decline. In the initial stage, the mass transfer flow rate is faster than in the middle time steps, and strong non-linear flow does not occur, indicating that prevention is crucial in the early stages. After the formation of the water influx channel, the velocity shows the strong non-linear characteristics, making the prevention more challenging. Therefore, in dealing with water influx disasters in the roof collapse column, it is essential to adhere to the principle of early detection and early intervention to prevent the evolution into more significant water inrush disaster.

## Data Availability

Some or all data, models, or codes generated or used during the study are available from the corresponding author by request.
